# The air conditioning in the nose of mammals depends on their mass and on their maximal running speed

**DOI:** 10.1038/s41598-024-59768-z

**Published:** 2024-04-20

**Authors:** Clément Rigaut, Alice Giaprakis, Laura Deruyver, Jonathan Goole, Pierre Lambert, Benoît Haut

**Affiliations:** 1 TIPs (Transfers, Interfaces and Processes), Université libre de Bruxelles, 1050 Brussels, Belgium; 2 Laboratoire de Pharmacie galénique et de Biopharmacie, Université libre de Bruxelles, 1050 Brussels, Belgium

**Keywords:** Air conditioning, Nose, Mammals, Fluid dynamics, Biological physics

## Abstract

The nose of the mammals is responsible for filtering, humidifying, and heating the air before entering the lower respiratory tract. This conditioning avoids, notably, dehydration of the bronchial and alveolar mucosa. However, since this conditioning is not perfect, exercising in cold air can induce lung inflammation, both for human and non-human mammals. This work aims to compare the air conditioning in the noses of various mammals during inspiration. We build our study on computational fluid dynamics simulations of the heat exchanges in the lumen of the upper respiratory tract of these mammals. These simulations show that the efficiency of the air conditioning in the nose during inspiration does not relate only to the mass *m* of the mammal but also to its maximal running speed *v*. More precisely, the results allow establishing a scaling law relating the efficiency of air conditioning in the nose of mammals to the ratio $v/\log _{10}(m)$. The simulations also correlate the resistance to the flow in the nose to the efficiency of this air conditioning. The obtained scaling law allows predicting the air temperature at the top of the trachea during inspiration for nasal-breathing mammals, and thus notably for humans of various ages.

## Introduction

The primary function of the mammalian respiratory system is the exchange of $\mathrm {O_2}$ and $\mathrm {CO_2}$ between the atmosphere and the blood^1^. Given the difference in size and fitness observed between animals, a natural conclusion is that the rate at which the body can harness oxygen in the air differs across the mammal class.

The respiratory system consists of two parts: the upper and the lower respiratory tracts. The upper tract includes the nose, the pharynx, and the larynx, while the lower respiratory tract spans from the trachea to the pulmonary alveoli^1^.

Besides handling the gas exchanges between the body and the environment, another crucial role of the respiratory tract is, during inspiration, to bring the air from ambient conditions to body temperature and full saturation in water vapor^2–4^. This conditioning is vital to avoid damage in the respiratory mucosa, triggering asthma^5^, inflammation^6,7^, and an increased risk of infections^8^. Notably, if inflammation occurs in the olfactory mucosa, there is a risk of (reversible) olfaction loss^9^. These problems arise, in particular, for athletes exercising in cold air. The nose is the first part of the respiratory tract that the air encounters during normal breathing and has an intricate anatomy accounting for 50 to 75 % of the total airway resistance to the flow of air^10^. Coherently, it is responsible for a large part of the air conditioning: *in-vivo* studies report that air entering the nose of an adult human breathing at room temperature and humidity (around 20 °C and 50 % relative humidity) exits this nose at a temperature between 31 and 34 °C, and with a relative humidity between 90 and 95 %^11^. It is due to the restricted tortuous passages of the nose, providing a large exchange surface to heat and moisten the air^11–16^. Despite the importance of air conditioning in the nose, only a few comparative studies aimed to explain the differences between individuals.

Moreover, to date, most of the studies devoted to air conditioning in the nose have focused only on humans^11,14–22^. While earliest works on this subject used simplified nose geometries to compute the temperature in the nose^16,17^, the recent studies used computational fluid dynamics (CFD) on realistic nose geometries to assess the temperature and humidity profiles of the air in the nose^14,15,21^. These simulations can even predict how the air conditioning would be affected by a surgical procedure^19,22^. On the other hand, only a few teams have studied non-human animals, and they have mainly focused on primates^23–25^. However, broadening the analysis from humans to the whole mammalian class would be interesting. First, mammals experience the same problems as humans when breathing cold air^26,27^. Second, we can use allometry (i.e. the dependence of physiology or anatomy on the body mass) to obtain more general laws. Indeed, allometric analysis studies have resulted in universal relationships to predict valuable parameters based on body mass^28,29^.

This work aims to gain insights into heat exchanges in the noses of terrestrial mammals during inspiration. The ability to predict the temperature at the end of the nose is interesting by itself but can also serve as input to evaluate the air temperature in the lungs. To this end, we compare the air conditioning in the nose of several mammals (including an adult and a young human) of various body mass (from 1.9 to 400 kg) and fitness, measured by their maximal running speed (from 21 to 70 $\mathrm {km \, h^{-1}}$). We intend to analyze if there are notable differences in the mechanisms involved, depending on the mass of the mammal (and also between fast mammals and those who are not), to identify the key parameters governing these heat exchanges, and to derive a scaling law that covers the broad spectrum of the terrestrial mammal mass, linking the physical characteristics of a mammal and its expected nose conditioning efficiency.

Our approach relies on CFD simulations based on the CT scan of the nose of various mammals, with diverse ambient temperatures and inspiratory flow rates. We use a fixed mucosal temperature of 32.6 °C^30^ to focus on the heat exchanges in the lumen of the airways. It is a simplification that does not include complex phenomena occurring on the mucosa (evaporative cooling, re-condensation of water at exhalation). However, incorporating these phenomena would require considering the heat exchanges in the tissues and the vascularisation of the mucosa^31–33^, which are still open research areas for most non-human mammals. Consequently, we only study here how heat transport in the lumen of the nose differs between mammals. Note that the mass diffusivity of water vapor in the air and the thermal diffusivity of the air have similar values, and both phenomena cool down the mucosa. Consequently, even if we only focus on heat transport in this article, it also gives clues about the transport of water vapour in the lumen of the noses of the mammals.

For this analysis, we select five non-human mammals covering a wide range of body mass and maximal running speed. Our selection consists of: a rabbit (1.9 kg) (*Oryctolagus cuniculus*), a cat (4 kg) (*Felis catus*), a dog (7 kg) (*Canis familiaris*), a snow leopard (60 kg) (*Panthera uncia*), and a horse (400 kg) (*Equus caballus*). We also include two humans of different ages: an adult (70 kg) and an 11-year-old child (38 kg). The mass associated with each mammal comes from Garland et al.^34^ and the Royal College of Paediatrics and Child Care^35^. We study the air conditioning in the nose of these seven mammals for ambient temperatures ranging from 5 °C to 30 °C, and for inspiratory flow rates corresponding to rest, low effort and moderate effort.

## Results and discussion

### Validation

We first compare the pressure-flow relationship obtained for the adult human with our simulations (see the Methods section) and the literature data to validate the flow simulations. This validation is also a first step in validating heat transport in the lumen of the nose since turbulent and thermal boundary layers have roughly the same thickness. We observe a difference of at most 5.5 % between our simulations and the data of Schroeter et al.^36^ (see Supplementary Figure S1). This difference is small enough to give us confidence about the computed flow given the variation in the anatomy of the nose across people.

Second, we validate the heat transport in the air. For that, we reproduce the boundary conditions of Naftali et al.^16^ to compare the temperature at the end of the nose obtained in our simulations for the adult human and their simulations. Their boundary conditions are an ambient temperature $T_i$ of 25 °C, a mucosa temperature $T_w$ of 37 °C, and an inspiratory flow rate *Q* of 15 $\mathrm {l} \, \mathrm{min}^{-1}$. The temperature at the end of the nose in their simulation is 35.3 °C while it is 35.5 °C in ours. Once again, the difference observed can be due to the anatomical differences in the nose.

### Air conditioning in mammals

**Figure 1:**
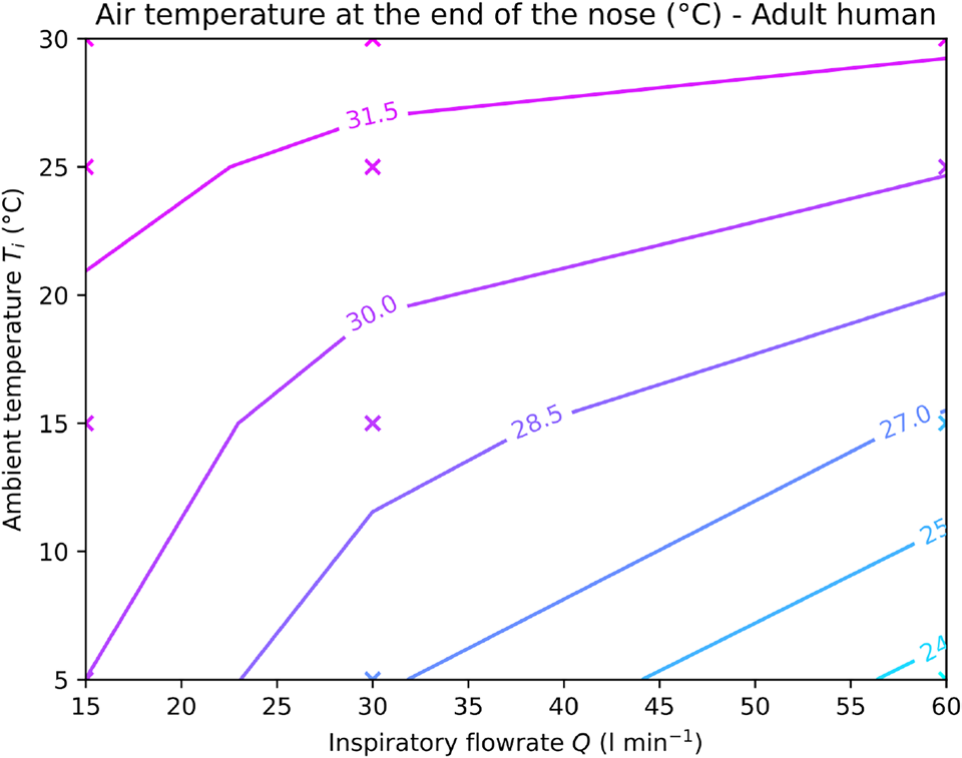
Temperature of the air at the end of the nose $T_o$ of the adult human, for an inspiratory flow rate ranging from 15 $\mathrm {l} \, \mathrm{min}^{-1}$ to 60 $\mathrm{l} \, \mathrm{min}^{-1}$, and for ambient temperatures $T_i$ ranging between 5 °C and 30 °C. Crosses represent the simulation points. Contours are linearly interpolated between these points.

With the simulations validated, we can now focus on varying the inspiratory flow rate *Q* (from rest to moderate effort) and the ambient air temperature $T_i$ (from 5 to 30 °C). Figure 1 shows the temperature at the end of the nose $T_o$ of the adult human as a function of the ambient temperature $T_i$ and of the inspiratory flow rate *Q*. The contour lines are obtained by linear interpolation of the temperature between the 12 simulated points (5 °C, 10 °C, 25 °C, and 30 °C, at rest, light effort, and moderate effort). As expected, the temperature of the air exiting the nose $T_o$ decreases with an increase of the inspiration flow rate *Q* and decreases with a decrease of the ambient temperature $T_i$. We can note that going from rest to moderate effort only decreases the temperature at the end of the nose by at most 4.8 °C (with an ambient temperature of 5 °C). On the other hand, switching the ambient temperature from 30 to 5 °C can decrease the air temperature at the end of the nose by 8.4 °C in the case of a moderate effort. This trend is the same for all mammals: the ambient temperature has more influence on air conditioning than the intensity of an effort does.

**Figure 2:**
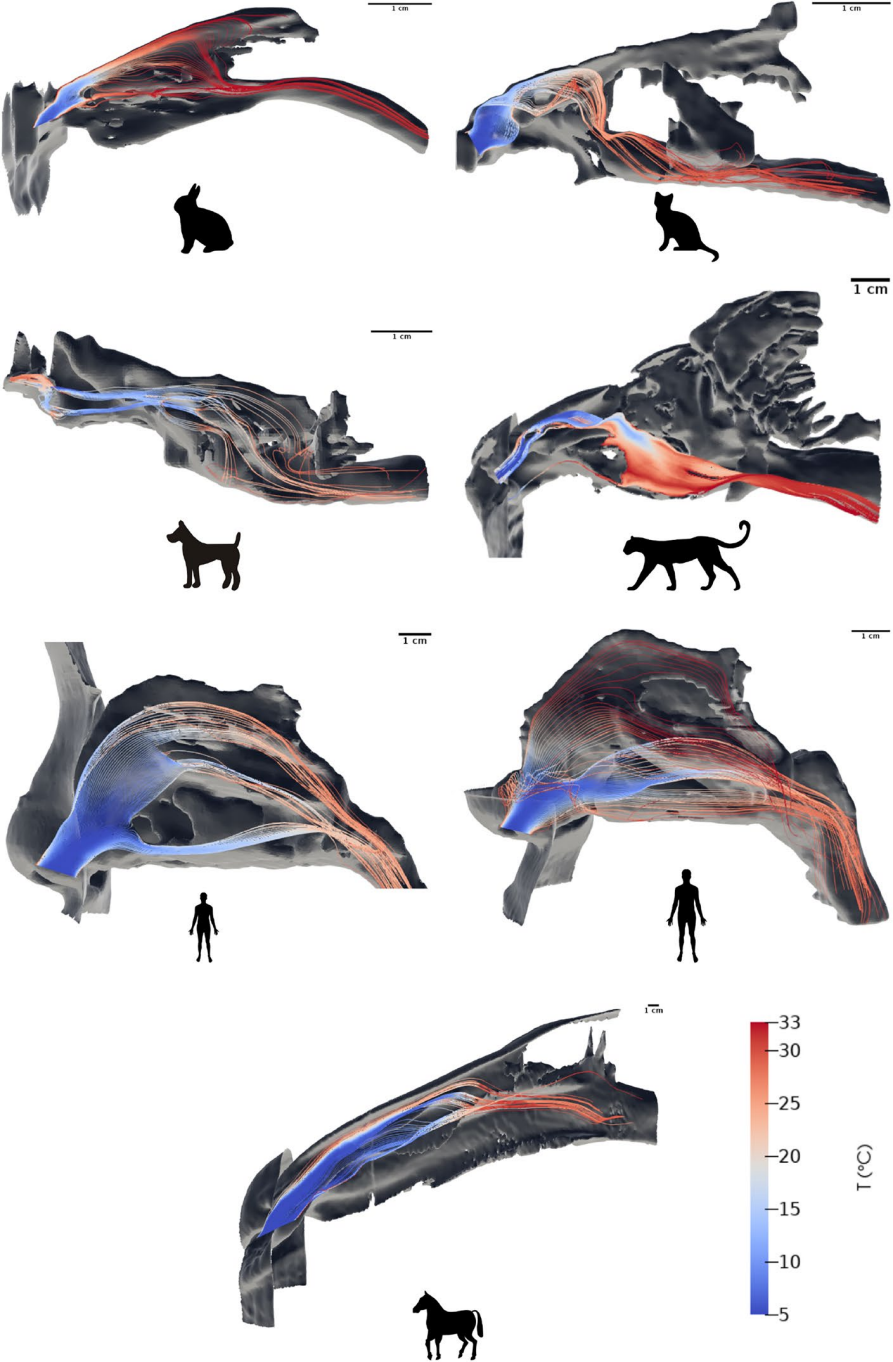
Streamlines of inspiratory airflow colored by air temperature, at moderate effort and for an ambient temperature of 5 °C, in the noses of the rabbit, the cat, the dog, the snow leopard, the 11-years-old human, the adult human and the horse. The nostrils of each mammal are on the left-hand side.

Figure 2 shows the inspiratory flow streamlines in the nose of the seven mammals considered, for an ambient temperature of 5 °C and at moderate effort. The streamlines are colored according to the air temperature and allow for appreciating qualitatively the differences between the mammals.

**Figure 3:**
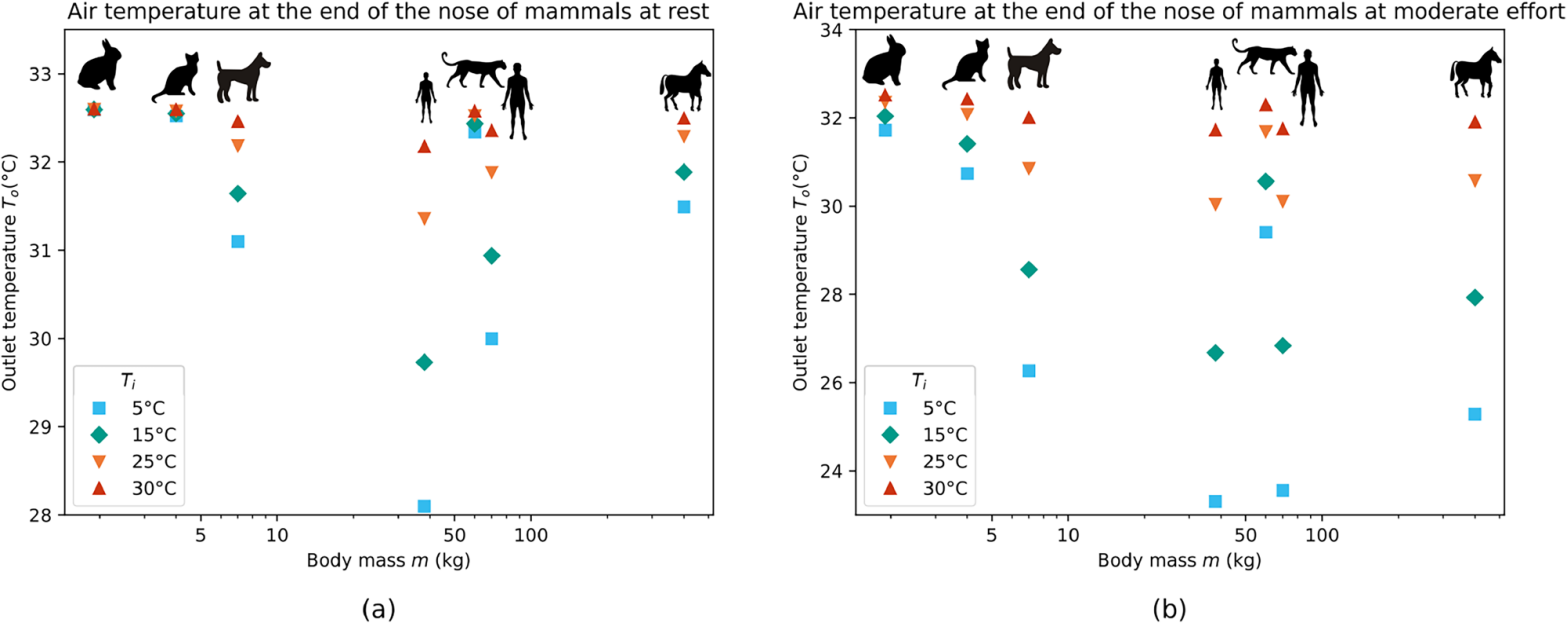
Temperature of the air at the end of the nose $T_o$ for the rabbit (1.9 kg), the cat (4 kg), the dog (7 kg), the 11-years old human (38 kg), the snow leopard (60 kg), the adult human (70 kg) and the horse (400 kg), at rest (**a**) or at moderate effort (**b**), and for ambient temperatures $T_i$ ranging between 5 and 30 °C.

It is tempting to combine the results of all the mammals by using their mass as a comparison basis. So, we represent the temperature at the end of the nose of each mammal $T_o$ as a function of its body mass *m*, for four ambient temperatures $T_i$, at rest (Fig. 3a) and at moderate effort (Fig. 3b). However, we can see that no clear trend appears in $T_o$ when doing this. This shows that there is no scaling law based only on the mass to characterize the air conditioning in the noses of mammals.

### Scaling law

To relate the air conditioning in the noses of the different mammals between them, one can suppose that it is needed to consider the fitness of the mammals. Indeed, it is natural to think that a mammal that can run fast has to condition air more effectively than a slow mammal. Table 1 shows the maximal running speed (used to measure fitness) and the mass of each studied mammal. Maximal running speeds for the cat, horse, rabbit, and adult human come directly from Garland et al.^34^. We define the maximal running speed of the dog as the average maximal speed of 7–8 kg dogs in the study of Haagensen et al.^37^. To determine the maximal running speed of an 11-years-old girl, we multiply her average running speed^38^ by the ratio between the maximal^34^ and the average^38^ running speeds for male adults. Table 1 shows that the mass of the mammals considered in this work spans over two orders of magnitude, while their maximal running speed spans over only one. So, we propose to use the logarithm of the mass to enable reasonable comparisons. It leads us to the definition of a parameter $\Lambda$ measuring the speed at which a mammal can run compared to its mass:

1 $$\begin{aligned} \Lambda = \frac{v/v_{\mathrm{ref}}}{\log _{10}(m/m_{\mathrm{ref}})} \end{aligned}$$

where *m* is the mass of the mammal in kg, *v* is its maximal running speed in $\mathrm {km} \, \mathrm{h}^{-1}$, $m_{\mathrm{ref}}$ is a reference mass, and $v_{\mathrm{ref}}$ is a reference maximal running speed, respectively equal to 1 kg and 1 $\mathrm {km} \, \mathrm{h}^{-1}$.

**Table 1:** Mass, maximal running speed, and $\Lambda$ $\left(\frac{v/v_{\mathrm{ref}}}{\log _{10}(m/m_{\mathrm{ref}})}\right)$ of the mammals used in this article.

Species	Body mass *m* (kg)	Maximal speed *v* ($\mathrm {km} \, \mathrm{h}^{-1}$)	$\Lambda$ (–)	References
Rabbit	1.9	56	201	^34^
Cat	4	40	66	^34^
Snow leopard	60	60	34	^34^
Horse	400	70	27	^34^
Dog	7	21	25	^37^
Human (adult)	70	40	22	^34^
Human (11 years)	38	33	21	^34,35,38^

**Figure 4:**
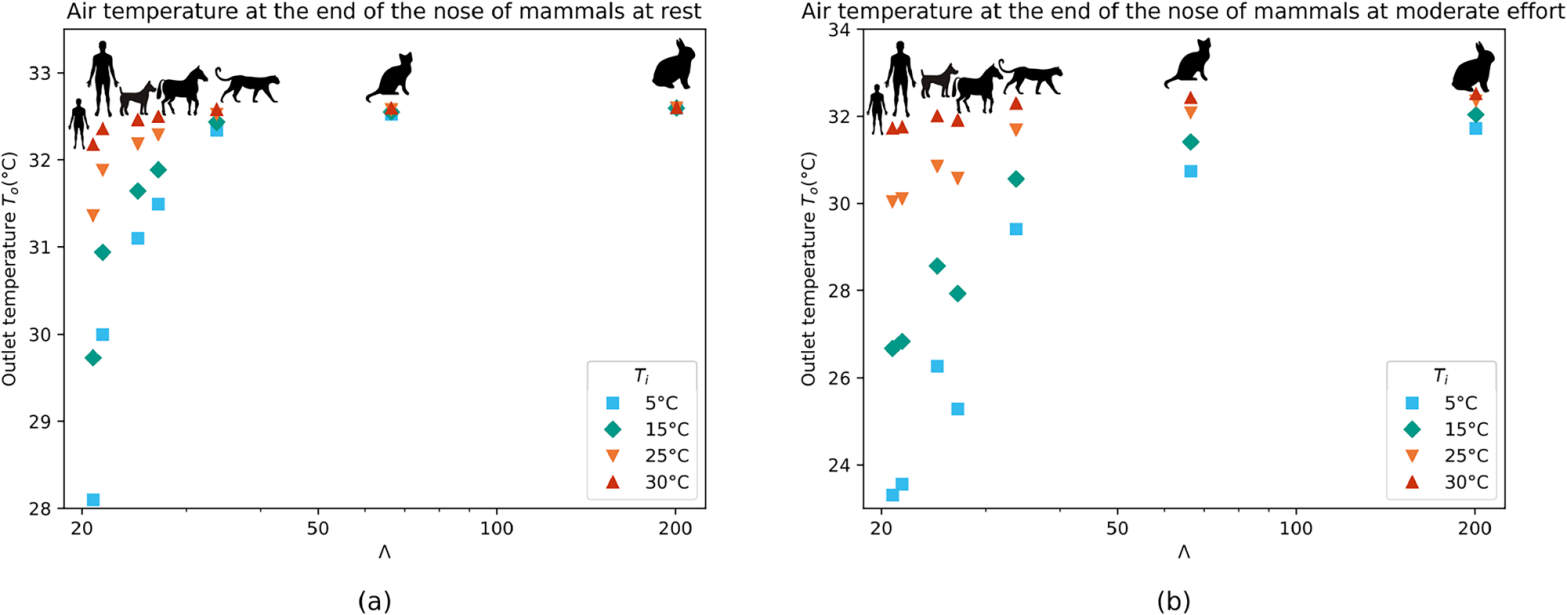
Temperature of the air at the end of the nose $T_o$, at rest (**a**) and at moderate effort (**b**), for ambient temperatures ranging between 5 and 30 °C, as a function of $\Lambda$, equal to the maximal running speed (in $\mathrm {km} \, \mathrm{h}^{-1}$) of a mammal divided by the logarithm of its mass (in kg).

This transformation emphasizes that the rabbits have a very high maximal running speed for their mass ($\Lambda = 201$) and that humans are poor runners given their relatively high body mass ($\Lambda = 22$ for the adult and $\Lambda = 21$ for the child). Then, we can plot the temperature at the end of the nose $T_o$ for each mammal at rest (Fig. 4a) and at moderate effort (Fig. 4b), as a function of the scaling parameter $\Lambda$. We see now a clear trend where, for the mammals presenting high values of $\Lambda$ such as the rabbit, $T_o$ is close to the mucosal temperature for every studied condition. On the other hand, for the frailest mammals (the young human), the temperature $T_o$ drops significantly and can be as low as 23 °C during a moderate effort in ambient air at 5 °C. This tendency supports the choice of a scaling parameter taking into account not only the mass but also the running speed of the mammal.

**Figure 5:**
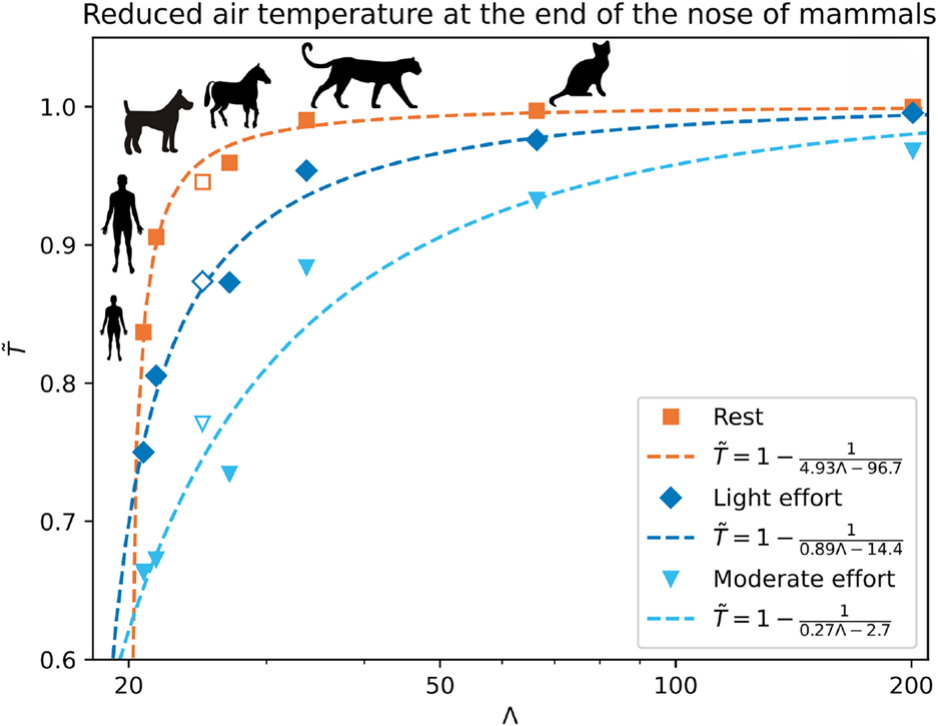
Reduced temperature of the air ${\tilde{T}}$ at the end of the nose as a function of $\Lambda$, at rest, light effort, and moderate effort. $\tilde{T} = \frac{T_o - T_i}{T_w - T_i}$, where $T_i$ is the ambient temperature, $T_o$ is the temperature at the end of the nose, and $T_w$ is the temperature of the mucosa. $\Lambda = \frac{v/v_{\mathrm{ref}}}{\log _{10}(m/m_{\mathrm{ref}})}$, where *v* is the maximal running speed of the mammal (in $\mathrm {km} \, \mathrm{h}^{-1}$) and *m* is its body mass (in kg). The empty symbols correspond to the dog. Dotted lines are the scaling laws for rest, light effort, and moderate effort. The last decimal kept in the coefficients of the laws correspond to the first significant digit in their error.

To study the air conditioning in the nose independently of the ambient temperature $T_i$ and the temperature of the mucosa $T_w$, we can define a non-dimensional parameter measuring the efficiency of air heating as the difference between the ambient temperature $T_i$ and the temperature at the end of the nose $T_o$, divided by the difference between the ambient temperature and the temperature of the mucosa $T_w$ : $\tilde{T} = \frac{T_o - T_i}{T_w - T_i}$. With this definition, ${\tilde{T}} = 0$ corresponds to no conditioning and ${\tilde{T}}=1$ to complete conditioning. Figure 5 shows ${\tilde{T}}$ as a function of $\Lambda$, at rest, during light effort, and at moderate effort. It appears that the dog (empty symbols) does not follow the trend of the other mammals (full symbols). It arises because rabbits, Felidae and horses are obligatory nasal breathers^39–41^, while humans, especially adults, switch from nasal to oronasal breathing when exercising^42^, and dogs are oronasal breathers even at rest^43^. This difference leads to an overestimation of the flow rate in the nose of the dog. Consequently, we remove the dog for the rest of our analysis.

In Fig. 5, we see again that the faster the mammal is, with respect to its mass (i.e. the larger $\Lambda$ is), the better its nose heats the air, both for rest and effort and that the difference between mammals increases with increasing effort. For mammals with $\Lambda$ above 30, the conditioning efficiency ${\tilde{T}}$ is always above 0.8, even at moderate effort. However, when $\Lambda$ is below 30, any decrease in its value is linked to a fall in the conditioning efficiency. For instance, between the adult ($\Lambda = 22$) and the child ($\Lambda = 21$), the conditioning efficiency drops by 7 %. In other words, there is a sudden decrease in $\tilde{T}$ around $\Lambda =30$. It suggests that $\Lambda$ cannot be much lower than 30 and that humans are among the worst runners in the mammalian class and not just in the studied panel. However, it is not so surprising because even elite humans are far from competing with the performance of mammals like horses or even dogs^44^.

Looking at the results presented in Fig. 5, we can now propose a scaling law to predict the air temperature at the end of the nose of a mammal (with $\Lambda >21$) under the form: ${\tilde{T}} = 1-(x\Lambda + y)^{-1}$. The coefficients *x* and *y* are determined by the least-squares method so that the scaling laws are the closest to the points given by the simulations for rest, light effort, and moderate effort. The result of this procedure is shown in Fig. 5. Such a law enables us to approximate the air temperature exiting the nose of a mammal for given respiratory conditions (flow rate and ambient temperature) by knowing only its mass and maximal speed. Also, since it is easy to determine mass and running speed for humans, one could use this law to estimate if adults or children risk damaging airways because of cold air in a given situation.

### Link with the nasal resistance to the flow

**Figure 6:**
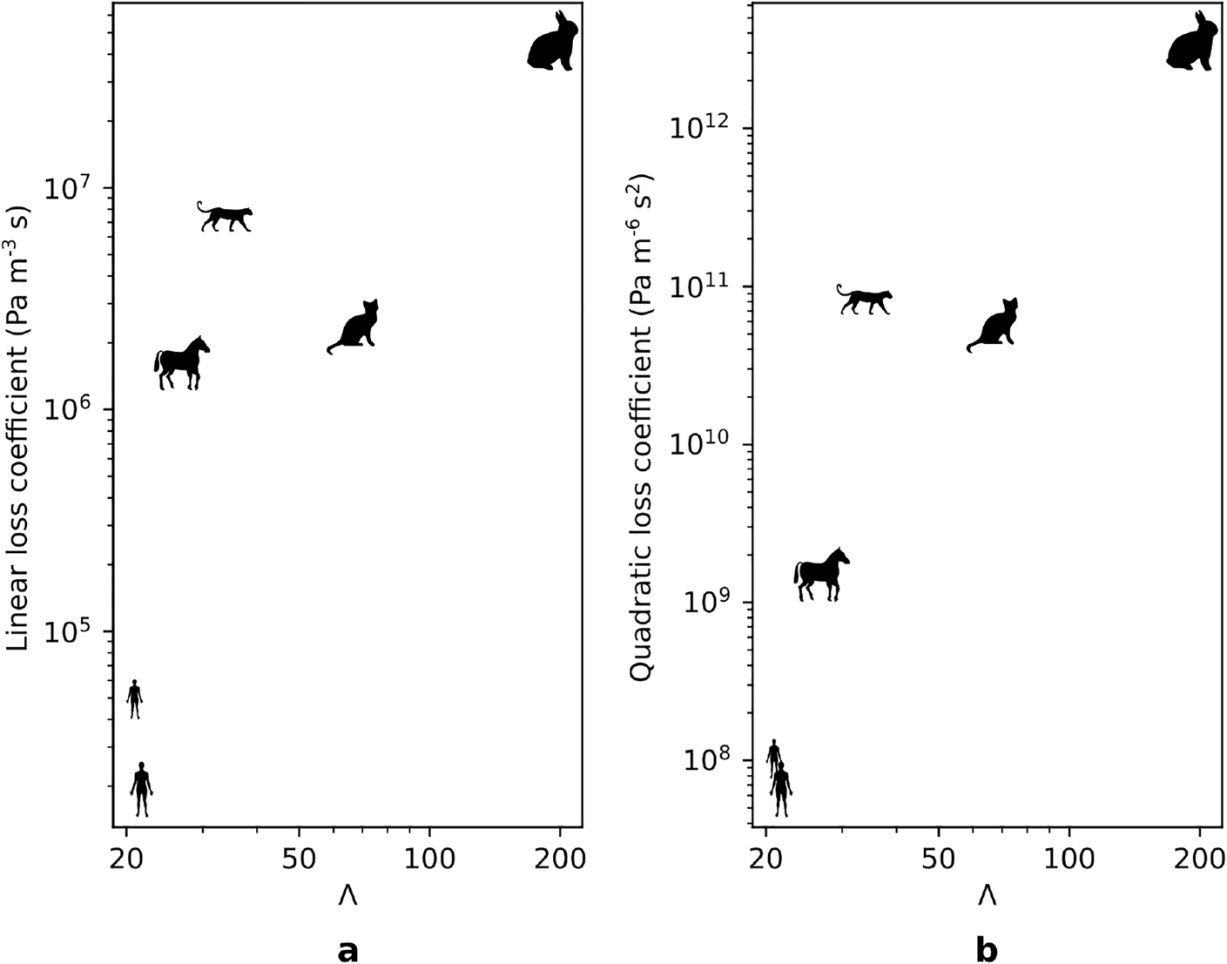
Linear (**a**) and quadratic (**b**) loss coefficients, obtained from the pressure-flow relationship of various mammals, as functions of $\Lambda = \frac{v/v_{\mathrm{ref}}}{\log _{10}(m/m_{\mathrm{ref}})}$, where *v* is the maximum running speed of the mammal (in $\mathrm {km} \, \mathrm{h}^{-1}$), *m* is its body mass (in kg), and $v_{\mathrm{ref}}$ and $m_{\mathrm{ref}}$ are reference mass and running speed, respectively.

As detailed in the Methods section, a pressure-flow relationship is derived for each mammal, relating the flow rate *Q* to the outlet pressure *p* (Supplementary Figure S2). Then, this relationship is fitted by a polynomial degree $|p| = aQ+bQ^2$, with *a* and *b* the linear and quadratic loss coefficients. Figure 6 presents *a* and *b* as functions of $\Lambda$.

Figure 6 shows that the faster the mammal is with respect to its mass (i.e. the higher $\Lambda$ is), the higher the linear and quadratic loss coefficients are. The clear relation between the quadratic loss coefficient and $\Lambda$ (Fig. 6b) reflects the link between the air conditioning and the inertial effects in the flow. Indeed, authors have highlighted the importance of inertial and turbulent flow structures in air conditioning for humans^18,45^. The present study shows that the inertial behavior of the flow is of the same importance for air conditioning in the nose for the entire mammalian class. It can be surprising to see a higher quadratic loss for mammals with a higher value of $\Lambda$. However, rabbits ($\Lambda = 201$) can run fast but over very short periods of time^46^ and snow leopards ($\Lambda = 34$) are more adapted to relatively short running periods^47^. On the opposite end of the spectrum, humans ($\Lambda = 22$) and horses ($\Lambda = 27$) naturally sustain lower-pace running for longer times^48^. It is also worth noting that the mammals with the highest $\Lambda$ (i.e. the cat and the rabbit) are also the smallest in the studied sample. So, the high resistance to the flow in their nose is due to the small cross-section of their nasal cavity.

## Conclusion

Air conditioning is a critical function of the nose for human and non-human mammals. Our study shows that not only the conditioning efficiency of a mammal is related to its mass *m*, but that its maximal running speed *v* plays a crucial role in this efficiency. More precisely, the results allow establishing a law relating the conditioning efficiency ${\tilde{T}}$ to $\Lambda = \frac{v/v_{\mathrm{ref}}}{\log _{10}(m/m_{\mathrm{ref}})}$, taking the form ${\tilde{T}} = 1 - \frac{1}{x\Lambda + y}$. The parameters *x* and *y* have been determined at rest, light effort, and moderate effort for $\Lambda >21$. This dependence on the running speed appears thanks to the broad sample of mammals studied and is not apparent only with humans. Also, with the inclusion of non-human mammals, we can see that, while the fast mammals (with respect to their mass) can almost fully condition the air in their nose, no matter the ambient temperature or their breathing rate, the slow ones have poor air conditioning during effort, with a sharp decrease of its efficiency for $\Lambda <30$. We also highlight that mammals with high values of $\Lambda$ tend to have high quadratic pressure loss in the nose, which correlates to excellent air heating in all conditions. On the other hand, slow mammals have lower quadratic pressure loss, leading to less efficient heating of the air in their nose.

The main limitation of the present study is to only consider the heat transport as a scalar, independent of water exchanges. Given the heat needed for evaporation and condensation of water, the wall temperature should not be constant and be allowed to vary. However, such boundary condition, which depends on the vascularisation of the nasal mucosa of each mammal, is beyond the scope of this article, and is open for future work.

## Methods

### 3D geometries

We start our study by processing the CT scan of the nose of each mammal. We obtained the scans of non-human noses from an open-source database^49^, and the two human scans were provided by the ENT department of the Erasme Hospital (Brussels, Belgium). All the methods were in accordance with relevant guidelines and regulations, notably to ensure the anonymity of the participants, as required by the Ethics Committee of the Erasme Hospital. Before starting the simulation process, we must obtain the shape of the cavity from the related CT scan. The first step is to transform the source DICOM file into a black-and-white image via a threshold on the grey value to separate the cavities from the body. The thresholded images are then cleaned up from isolated white or black pixels by two successive morphological operations: an opening followed by a closing, both using a 3 × 3 square structuring element^50^. At this stage, we remove the sinuses since they have a minute impact on nasal airflow^51^. We repeat this procedure for all slices composing the full scan to obtain the 3D geometry. However, the surface obtained is rough due to the limited resolution of the scans. A final step is thus to smooth the resulting 3D model to have a ready-to-use STL file. A Laplacian smoothing is applied until the surface/volume ratio does not change more than 1% between two successive iterations. Finally, we generate the computation mesh based on this file. The STL files are available for download in the supplementary material.

### Flow equations and boundary conditions

The air in our simulations is a non-compressible Newtonian fluid. This assumption comes from the expected low maximal velocity, leading to low Mach numbers, which is indeed confirmed by the simulation results. The thermal diffusivity of the air is fixed at $\alpha = 22 \times 10^{-6}~\mathrm{m}^2 \, \mathrm{s}^{-1}$, its value at 20 °C^52^. This assumption should not impact the results given the low dependence of $\alpha$ to the temperature in the range considered in this study.

Martonen et al. showed that laminar flow models cannot fully capture the flow in the entire nose^53^. The turbulence model selected for our simulations is the $\mathrm {k -} \omega$ Shear Stress Transport (SST) model. Indeed, the air passing through the nose encounters various cross-sections, leading to a wide range of Reynolds numbers^54^. Additionally, the small size of the airways imposes a fine mesh with near-to-wall cells too thin to apply a wall function, which implies resolving the flow in the laminar sub-layer^55^. The SST $\mathrm {k -} \omega$ model is thus suitable to study the flow in the nose^56^. As reported by Schillaci and Quadrio, the results obtained using an SST $k-\omega$ model differ only slightly from the ones obtained using Large Eddy Simulations (LES)^57^. More precisely, the centre of the nasopharynx is the only area with significant velocity differences between both approaches (and thus, on the contrary, the velocity fields in the turbinates predicted by both approaches are very close to each other). So, the extent of air conditioning should be the same either by using an SST $k-\omega$ model or by using LES, as the flow in the turbinates is mainly responsible for this air conditioning. Also, for the smallest mammals, especially at low inspiratory flow rate, the data in Supplementary Table S1 show that the flow in the nose is fully laminar. However, Supplementary Table S2 shows that our simulations using the SST $k-\omega$ turbulence model do not show significant differences in air conditioning with full laminar simulations we also performed for the rest situation. This result is also in line with the work of Schillaci and Quadrio, who observed similar pressure-flow relationships in the nose between laminar and SST $k-\omega$ simulations^57^.

Those hypotheses give the following equations to describe momentum and heat transport in the lumen of the nose^58^:

2 $$\begin{aligned}& \boldsymbol{\nabla } \cdot {\textbf{u}} = 0 \end{aligned}$$

3 $$\begin{aligned}&\frac{\partial {\textbf{u}}}{\partial t} + ({\textbf{u}} \cdot \boldsymbol {\nabla }) {\textbf{u}} = -\boldsymbol {\nabla } p + \boldsymbol {\nabla } \cdot ((\nu + \nu _t) \boldsymbol {\nabla}\mathbf{u}) \end{aligned}$$

4 $$\begin{aligned}&\nu _\mathrm{t} = \frac{k}{\omega } \end{aligned}$$

5 $$\begin{aligned}&\frac{\partial k}{\partial t} + {\textbf{u}} \cdot \boldsymbol {\nabla } k = \boldsymbol {\nabla } \cdot (D_{k} \boldsymbol {\nabla } k) + G - \frac{2}{3} k (\boldsymbol {\nabla } \cdot {\textbf{u}}) - \beta ^* \omega k \end{aligned}$$

6 $$\begin{aligned}&\frac{\partial \omega }{\partial t} + {\textbf{u}} \cdot \boldsymbol {\nabla } \omega = \boldsymbol {\nabla } \cdot (D_{\omega } \boldsymbol {\nabla } \omega ) + \frac{\gamma G}{\nu } - \frac{2}{3} \gamma \omega (\boldsymbol {\nabla } \cdot {\textbf{u}}) - \beta \omega ^2 - (F - 1) CD_{k\omega } \end{aligned}$$

7 $$\begin{aligned}&\frac{\partial T}{\partial t} + {\textbf{u}} \cdot \boldsymbol {\nabla } T = \boldsymbol {\nabla } \cdot ((\alpha + \alpha _t) \boldsymbol {\nabla } T) \end{aligned}$$

where ${\textbf{u}}$ is the air velocity vector, *p* is the pressure, *T* is the temperature, $\nu$ is the kinematic viscosity of the air, $\nu _t$ is the eddy viscosity, *k* is the turbulent kinetic energy, $\omega$ is the turbulence frequency, $D_k$, $D_{\omega }$, and $CD_{k \omega }$ are the effective diffusivities of *k*, $\omega$, and the cross-diffusivity of *k* and $\omega$, respectively, *G* is the turbulent kinetic energy production rate due to the asymmetric part of the stress tensor, *F* is the model blending function, $\alpha$ is the thermal diffusivity of the air, $\alpha _t$ is the turbulent thermal diffusivity, the rest of the symbols being model constants. All the parameters are considered independent of the temperature and calculated at 20 °C. A summary of their values is presented in Table 2.

The boundary conditions used in our simulations are no-slip condition and fixed temperature on the mucosa $T_w$, fixed pressure ($p=0$) and temperature $T_i$ and no velocity gradient at the inlet (i.e. the nostrils), and no pressure and temperature gradients with fixed flow rate *Q* at the outlet. We set the outlet as the cross-section of the lumen containing the top of the soft palate. As mentioned in the introduction, the temperature of the mucosa is set to 32.6 °C for all the mammals (it is the mean temperature of the nasal mucosa reported by Lindemann et al.)^30^. We perform the simulations by solving transient equations with a constant value of *Q* until a stationary state is reached.

**Table 2:** Values of the parameters used in the simulations.

Parameter	Symbol	Value at 20 °C	Unit
Kinematic viscosity of air	$\nu$	$1.5 \times 10^{-5}$	$\mathrm {m}^2 \, \mathrm{s}^{-1}$
Thermal diffusivity of air	$\alpha$	$2.2 \times 10^{-5}$	$\mathrm {m}^2 \, \mathrm{s}^{-1}$
Closure coefficient in the turbulent-kinetic-energy equation	$\beta ^*$	0.09	–
Closure coefficient in the specific dissipation-rate equation	$\gamma$	5/9 (bulk); 0.44 (walls)	–
Closure coefficient in the specific dissipation-rate equation	$\beta$	0.075 (bulk); 0.0828 (walls)	–

### Inspiratory conditions

For all the mammals, we consider three temperatures of the ambient air $T_i$: 5 °C, 20 °C, and 30 °C. Because of the fixed temperature of the mucosa, our model cannot consider ambient temperatures above 32.6 °C. We thus decide to limit the ambient temperature to at most 30 °C.

For each mammal, we study three inspiratory flow rates *Q* corresponding to rest breathing, light effort, and moderate effort. Higher efforts are not considered since humans switch from nasal to oro-nasal breathing at sustained effort^42^. For the adult human with a mass $m = m_{\mathrm{ref}} = 70\ \mathrm{kg}$, $Q = 15\ \mathrm {l}\, \mathrm{min}^{-1}$ at rest, $Q = 30\ \mathrm {l}\, \mathrm{min}^{-1}$ at light effort and $Q = 60\ \mathrm {l} \, \mathrm{min}^{-1}$ at moderate effort. To evaluate these three flow rates for a mammal of mass *m*, the values for the adult human are multiplied by $\left( \frac{m}{m_{\mathrm{ref}}}\right) ^{\frac{3}{4}}$ (adapted from Lindstedt and Schaeffer’s law for rest breathing: $Q=7.72\ \mathrm{m}^{0.745}$ where *Q* is in ml/s and *m* in kg^59^). Supplementary Table S3 summarizes the different values of the inspiratory flow rates used in the simulations. In each case, we use steady-state inspiratory conditions. If we assume symmetrical inspiration and expiration, the inspired volume per minute is half our inspiratory flow rate in $\mathrm {l} \, \mathrm{min}^{-1}$.

### Numerical methods

#### Mesh

The meshes used for the simulations are hexahedral meshes with two wall layers. We select the thickness of these layers to avoid an abrupt transition from the bulk and a $y^+$ lower than 1, as required for the $\mathrm {k -} \omega$ SST turbulence model. We also test the quality of the meshes to ensure there is no cell with excessive skewness, aspect ratio, or non-orthogonality.

We generate four different meshes, numbered from 1 to 4, for the adult human, with around 800,000, 1,900,000, 3,500,000, and 4,500,000 cells. We carry out test simulations with multiple inspiratory flow rates in this geometry. The maximal velocity difference observed between meshes 2 and 3 was around 3 %. Additionally, we vary the number of wall layers and their thickness while keeping $y^+$ below 1. We observe no significant difference neither between meshes 2, 3 and 4 nor for meshes with more than two wall layers. So, we apply the parameters used to generate mesh 2 to create all our simulation meshes.

To transpose the parameters from the mesh of the adult human to the other meshes, we keep the same base cell size and refine the mesh until the thickness of the outermost wall layer complies with the constraint $y^+ < 1$. A cross-section of each mesh, taken in the middle of the turbinates, is shown in Supplementary Figure S3.

#### Solvers and discretization schemes

We use the PISO algorithm for pressure-flow coupling with a multigrid Gauss–Seidel solver for the pressure equation and a bi-combined gradient solver with an incomplete LU-factorization pre-conditioner for the momentum and the turbulence equations. The discretization schemes used are Crank–Nicolson for the time, linear upwind for the velocity, and central differencing for the other variables. The time steps are adjusted to keep the Courant number below 1 for all cells.

### Post-processing

For each mammal and each inspiratory condition, we compute the temperature at the end of the nose $T_o$ by averaging the local temperature at the outlet:

8 $$\begin{aligned} T_o = \frac{1}{Q} \iint _{\Omega } T\ {\textbf{u}} \cdot {\textbf{n}} \ \mathrm{d}S \end{aligned}$$

where ${\textbf{n}}$ is the unit vector normal to the outlet.

To analyze the pressure drop through the nose during inspiration, we draw the “pressure-flow” relationship of each mammal by computing the outlet pressure *p* for five values of *Q*: $0.5 Q_{\mathrm{rest}}$, $Q_{\mathrm{rest}}$, $2Q_{\mathrm{rest}}$, $3Q_{\mathrm{rest}}$, and $4Q_{\mathrm{rest}}$, with $Q_{\mathrm{rest}}$ the inspiratory flow rate of the mammal at rest. We then fit a polynomial $|p| = aQ+bQ^2$ to the numerical values, where *a* and *b* are the linear and quadratic loss coefficients.

### Validation

To ensure that the velocity and the pressure given by our simulations are in line with the literature, we compare the pressure-flow relationship for the adult human obtained with our simulations and the one of Schroeter et al.^36^. Moreover, we set the temperatures obtained with our simulations against the values reported by Naftali et al.^16^ for the adult human to validate scalar transport.

### Ethical approval

 Human scans were obtained from Erasme Hospital—ULB. The study was reviewed and approved by the Ethics Committee of Erasme Hospital—ULB. The Ethics Committee waived the requirement of written informed consent for participation. Animal scans are freely available under Creative Commons licence CC-BY 4.0 (rabbit: https://www.embodi3d.com/files/file/14319-rabbit-skull/; cat: https://www.embodi3d.com/files/file/45885-hester-louie/; dog: https://www.embodi3d.com/files/file/44221-biggie; snow leopard: https://www.embodi3d.com/files/file/41744-snow-leopard-test/; horse: https://www.embodi3d.com/files/file/18571-horse-head).

### Supplementary Information

Supplementary Information.

## Data Availability

All data generated or analyzed during this study are included in this published article (and its Supplementary Information files).
