# Supplementary material for: The air conditioning in the nose of mammals depends on their mass and on their maximal running speed
Source: Sci Rep. 2024 Apr 20;14:9053. doi: 10.1038/s41598-024-59768-z (PMC11032399; doi:10.1038/s41598-024-59768-z)
Supplement: Supplementary file 1 — Supplementary Information. [file 41598_2024_59768_MOESM1_ESM.pdf]

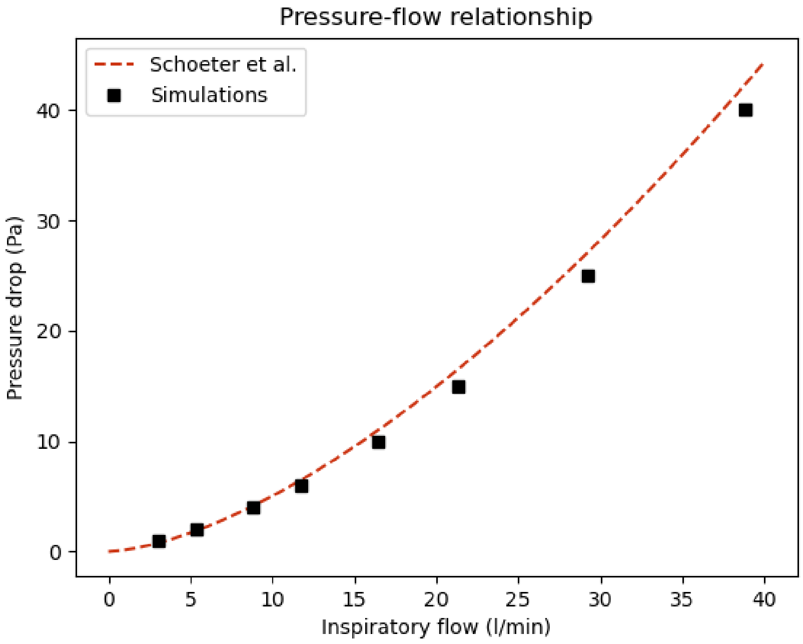


Figure S1: Pressure-flow relationship obtained with our simulations for the adult human and by Schroeter et al.

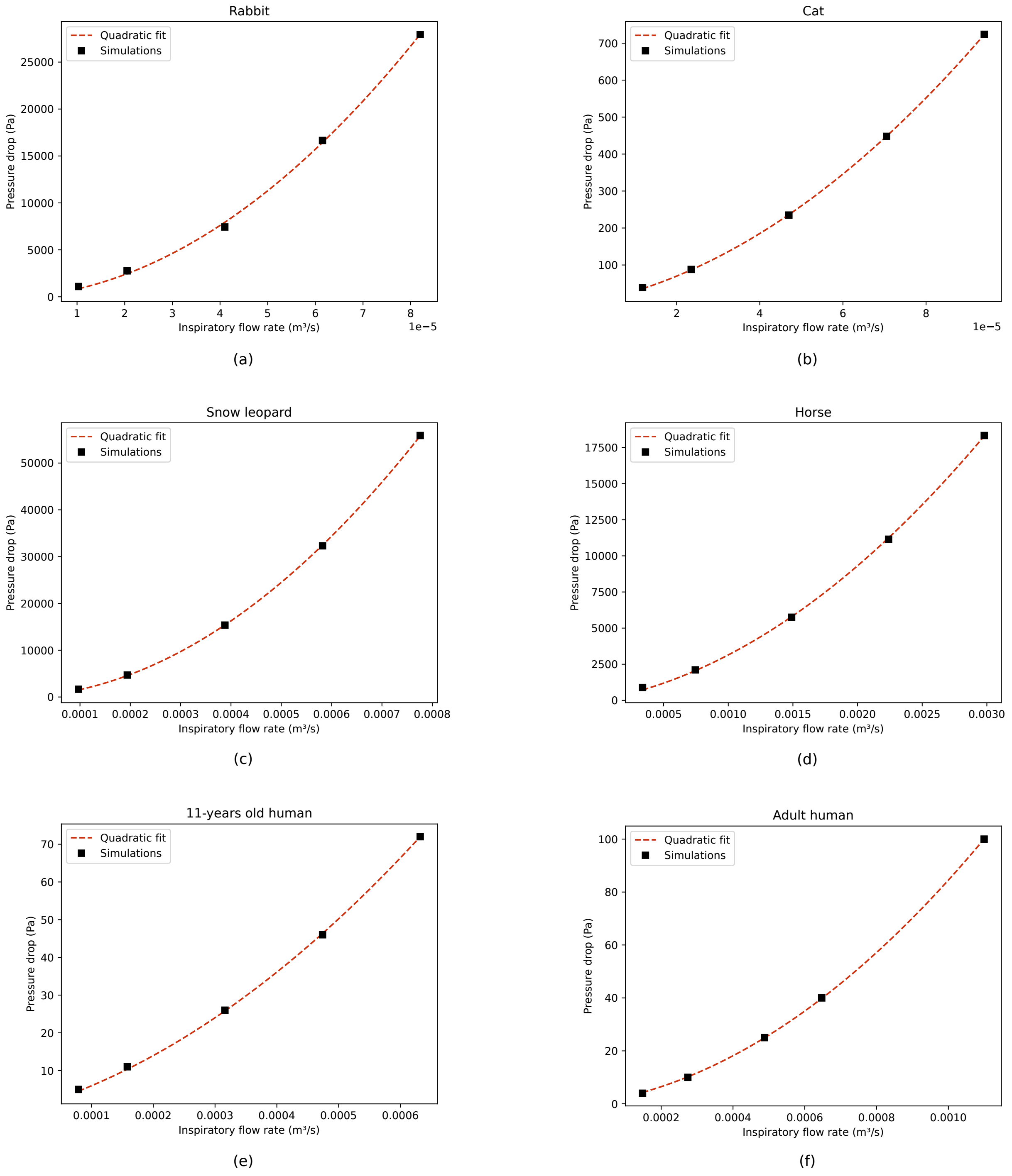


Figure S2: Pressure-flow relationships of the nose-breathing mammals used in this article.

| | $\Lambda(-)$ | Air velocity ($\mathrm{m \cdot s^{-1}}$) | Re (-) |
|---|---|---|---|
| Rabbit | 201 | 5.47 | 881 |
| Cat | 66 | 4.61 | 607 |
| Snow leopard | 34 | 9.05 | 1923 |
| Horse | 27 | 2.07 | 2628 |
| Dog | 25 | 4.01 | 942 |
| Human (adult) | 22 | 5.75 | 3106 |
| Human (11 years) | 21 | 4.77 | 2299 |

Table S1: $\Lambda$ values of the mammals used in this article, mean velocities of the air in the nasal valves at moderate effort, and corresponding Reynolds numbers.

| | $\tilde{T}$ (laminar) | $\tilde{T}$ (SST $k-\omega$) |
|---|---|---|
| Rabbit | 0.9982 | 0.99971 |
| Cat | 0.99768 | 0.999714 |
| Snow leopard | 0.99425 | 0.99052 |
| Horse | 0.96994 | 0.95980 |
| Dog | 0.94563 | 0.94559 |
| Human (adult) | 0.90836 | 0.90567 |
| Human (11 years) | 0.83463 | 0.83691 |

Table S2: Reduced temperatures of the air $\tilde{T}$ at the end of the nose computed at rest, with a full laminar model and using the turbulent SST $k - \omega model$.

| | Rest ($\mathrm{m^3 \cdot s^{-1}}$) | Light effort ($\mathrm{m^3 \cdot s^{-1}}$) | Moderate effort ($\mathrm{m^3 \cdot s^{-1}}$) |
|---|---|---|---|
| Rabbit | $2.05\ 10^{-5}$ | $4.10\ 10^{-5}$ | $8.20\ 10^{-5}$ |
| Cat | $2.35\ 10^{-5}$ | $4.70\ 10^{-5}$ | $9.40\ 10^{-5}$ |
| Snow leopard | $1.94\ 10^{-4}$ | $3.88\ 10^{-4}$ | $7.76\ 10^{-4}$ |
| Horse | $7.45\ 10^{-4}$ | $1.49\ 10^{-3}$ | $2.98\ 10^{-3}$ |
| Dog | $5.80\ 10^{-5}$ | $1.16\ 10^{-4}$ | $2.32\ 10^{-4}$ |
| Human (adult) | $2.50\ 10^{-4}$ | $5.00\ 10^{-4}$ | $1.00\ 10^{-3}$ |
| Human (11 years) | $1.58\ 10^{-4}$ | $3.16\ 10^{-4}$ | $6.32\ 10^{-4}$ |

Table S3: Inspiratory flow rates at rest, light effort and moderate effort of the mammals used in this article.

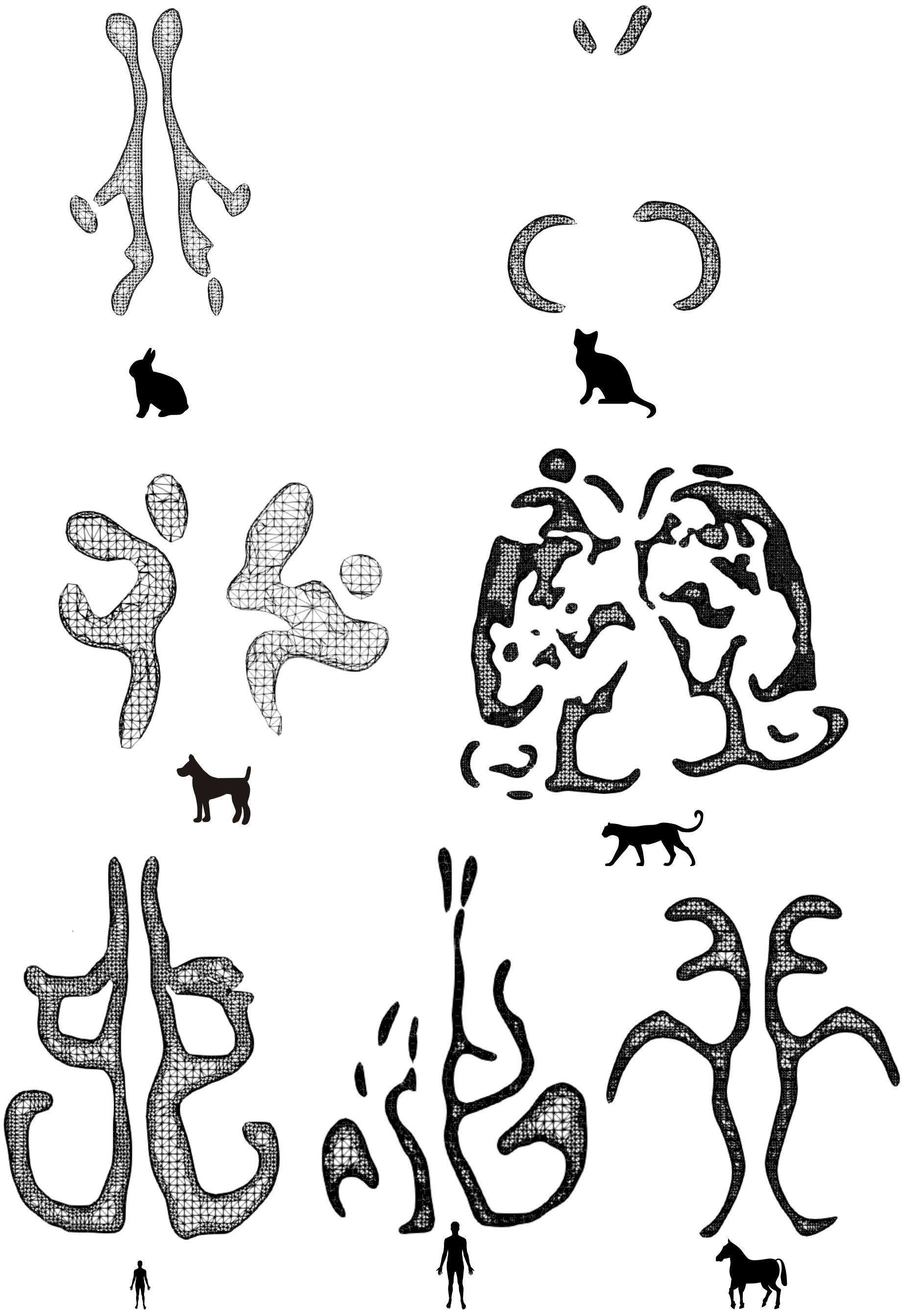

Figure S3: Frontal sections of the meshes used in this article.
